# Effects of the Intermittent Theta Burst Stimulation of the Cerebellar Vermis on Balance Recovery After Stroke: A Study Protocol for a Randomized Controlled Trial

**DOI:** 10.3389/fnagi.2022.881311

**Published:** 2022-04-29

**Authors:** Lin Wang, Guilan Huang, Li Zhang, Jinyu Yang, Caili Ren, Chengpan Liang, Ying Shen, Bin Su

**Affiliations:** ^1^School of Kinesiology, Shanghai University of Sport, Shanghai, China; ^2^Department of Rehabilitation, Wuxi Tongren Rehabilitation Hospital, Wuxi, China; ^3^Department of Neurorehabilitation, Wuxi Tongren Rehabilitation Hospital, Wuxi, China; ^4^Rehabilitation Medicine Center, The First Affiliated Hospital of Nanjing Medical University, Nanjing, China

**Keywords:** study protocol, stroke, intermittent theta burst stimulation, cerebellar vermis, RCT

## Abstract

**Background:**

The recovery of balance function is a critical segment in the rehabilitation treatment of stroke. The cerebellum is considered as the key structure involved in balance and motor control. The cerebellar vermis plays an important role in integrating vision, proprioception, and sensory skin input and may be a candidate stimulation target for regulating the motor network related with balance. However, evidence that the intermittent theta burst stimulation (iTBS) of cerebellar vermis can promote the recovery of balance function after stroke remains insufficient. Therefore, this study aims to explore the efficacy of the cerebellar vermis iTBS for the treatment of balance function in patients with stroke.

**Methods and Analysis:**

Forty patients with stroke will be recruited in this prospective, randomized, sham-controlled trial. Participants will be randomized in a 1:1 ratio to receive either 15 sessions of cerebellar vermis iTBS (600 pulses) or sham stimulation. Additionally, a routine rehabilitation therapy follows the intervention. The primary outcome is the Berg Balance Scale, and the secondary outcomes are the Fugl–Meyer assessment of the lower extremity and modified Barthel index. The above outcomes will be assessed before intervention and at the end of each week. Pre- and post-iTBS resting-state functional magnetic resonance imaging (rs-fMRI) will be acquired, and the regional homogeneity, fractional amplitude of low-frequency fluctuation and functional connectivity will be calculated and analyzed.

**Discussion:**

This protocol holds promise as a potential method to improve balance function in patients with stroke. If the outcomes of patients improve after the intervention, the study will provide new insights into improving balance function.

**Ethics and Dissemination:**

This study has been approved by the Medical Research Ethics Committee of Wuxi Mental Health Center (Wuxi Tongren Rehabilitation Hospital). Results will be disseminated through (open-access) peer-reviewed publications, networks of scientists, professionals, and the public and presented at conferences.

**Clinical Trial Registration Number:**

www.chictr.org.cn, identifier ChiCTR2100052590.

## Introduction

Every year, over 15 million people in the world suffer from stroke (Van Lieshout et al., 2017). Balance problem is a common dysfunction that arises after stroke and is usually due to poor proprioception, decreased motor control, and abnormal integration of nervous system (Xiong et al., 2019). Moreover, the balance problem may be the main cause of poor walking ability and increased risk of falls in patients with stroke (Zhang et al., 2020). About 15–37% of patients with stroke fall down at least once due to balance dysfunction during hospitalization (Rabadi et al., 2012). Falls appear to be frequent after discharge, with approximately 40% falling within 6–12 months of stroke (Wada et al., 2007). Therefore, balance plays a significant role in the rehabilitation process of stroke. However, pharmacotherapy (Mead et al., 2013) and conventional rehabilitation treatments, including core strength exercises (Chung et al., 2014), visual feedback training (Zhang et al., 2020), neurodevelopmental therapy (Langhammer and Stanghelle, 2011), and proprioceptive neuromuscular facilitation (Eng and Tang, 2007) performed unsatisfactory results on balance recovery among stroke patients.

In recent years, repetitive transcranial magnetic stimulation (rTMS), a non-invasive brain stimulation technology, has been widely used in various nervous system diseases (Ren et al., 2019; Yin et al., 2020). rTMS induces a current in the cerebral cortex through a coil that generates a magnetic field, thereby regulating the excitability of the cerebral cortex and affecting neuroplasticity (Brown et al., 2021). Theta burst stimulation (TBS), a novel pattern of rTMS, has the characteristics of lower stimulation intensity and a shorter stimulation session (2–3 min or less) (Pinto et al., 2018), the relatively low stimulation intensity reduces the risk of adverse reactions especially the risk of epileptic seizures (Gutierrez-Muto et al., 2020). Huang and Rothwell (2004) were the first to investigate the neurophysiological effects of TBS in healthy human subjects, founded that 600-pulse intermittent TBS (iTBS) can enhance cortical excitability.

The cerebellum plays an important role in maintaining postural position, regulating the muscle tension associated with postural movements, and coordinating voluntary movements (Manto et al., 2011). To date, studies on the effects of cerebellar rTMS on balance rehabilitation are still limited. As pointed out in neurophysiological research, the cerebellar activity in patients with stroke is positively correlated with the recovery of walking function (Bijsterbosch et al., 2011). Low-frequency rTMS in the cerebellum can improve walking ability in patients with spinocerebellar degeneration (Svenja et al., 2017). Furthermore, a meta-analysis suggested that cerebellar stimulation may affect corticospinal excitability (Zelda et al., 2016). Koch et al. (2018) also found that the iTBS of the unaffected cerebellum promotes the excitability of the posterior parietal cortex and improves the balance and gait function in patients with chronic stroke (over 6 months).

Notably, the choice of the rTMS target has a remarkable influence on its therapeutic effect. However, an agreement on how to select the best targets for patients with balance dysfunctions is limited until now. Primary motor cortex (M1) area is one of the most common targets. Kim et al. (2014) used low-frequency rTMS to act on the M1 area of the patient’s lower extremities and found that the 10 min walking speed and Berg Balance Scale (BBS) are improved. Additionally, the supplementary motor area (SMA) (Richard et al., 2017) and dorsal prefrontal cortex (Harris et al., 2018) have been implicated to be critical in balance tasks. With deepening research, recent studies demonstrated that the cerebellum is also one of the effective targets of rTMS. The cerebellar vermis is a major part of the cerebellum. Notably, patients with cerebellar vermis lesions present with balance dysfunction (Harris et al., 2018), whereas cerebellar hemisphere lesions focus on global coordination dysfunctions (Carass et al., 2018; Maas et al., 2020). A recent study that used fNIRS technology found that after a single iTBS on the cerebellar vermis in healthy individuals, the HBO_2_ values in bilateral SMA regions are increased. These results indicated a potential link between the cerebellar vermis and SMA (Tan et al., 2021). The cerebellar vermis may be a new target for iTBS to intervene with balance function and deserves further exploration.

A randomized controlled clinical trial will be conducted to investigate the rehabilitation effect of the cerebellar vermis intervened by iTBS in stroke patients with balance dysfunctions. Furthermore, the resting-state functional magnetic resonance imaging (rs-fMRI) will be used to analyze the functional connections between cerebellar vermis and other areas of the cerebral cortex, and clarify the neural remodeling mechanism. We propose a hypothesis that iTBS of cerebellar vermis can promote the recovery of balance function after stroke. This study may provide new insights into the therapeutic efficacy and underlying neuroplasticity mechanisms of cerebellar vermis intervention.

## Materials and Methods

### Study Design

This study will be carried out in the Department of Rehabilitation, Tongren Rehabilitation Hospital, Wuxi, China. Before the conduct of formal experiments, a research coordinator who is responsible for recruitment will arrange a conversation with potential participants to discuss some details, including research purpose, procedures, and the potential risks and benefits. Once informed consent is signed by the patient or their legal guardians (if the patient cannot write independently or consider consent), a questionnaire and baseline assessment will be arranged.

**FIGURE 1:**
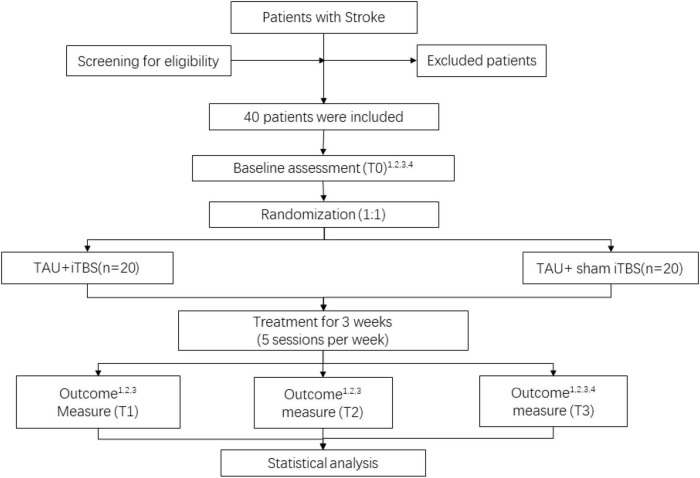
Study flow diagram. ^1^BBS, ^2^FMA-LE, ^3^MBI, and ^4^fMRI. TAU, treatment as usual; iTBS, intermittent theta burst stimulation. T0, before intervention; T1, at the end of first week of post-intervention; T2, at the end of second week of post-intervention; T3, at the end of third week of post-intervention.

All participants will be randomly assigned to real or sham iTBS and then receive routine rehabilitation treatment, and they will be assessed using the BBS, Fugl–Meyer assessment of the lower extremity (FMA-LE), and modified Barthel index (MBI). Moreover, rs-fMRI scans will be performed at baseline and 3 weeks after treatment. The study flow chart is shown in Figure 1. All baseline data gathering, follow-up assessment, and interventions will be carried out by well-trained researchers with the support of academic staff. This study has been approved by ethics committees (WXMHCIRB2021LLky132) and registered on www.chictr.org.cn on 27 October 2021 under the number ChiCTR2100052590. Details are shown in Table 1.

**TABLE 1:** Trial registration data.

Data category	Trial information
Primary registry and trial identifying number	ChiCTR2100052590
Date of registration in primary registry	27 October 2021
Secondary identifying numbers	N/A
Source(s) of monetary or material support	This project is being supported by Wuxi Taihu Talent Project (WXTTP2020008), the Nanjing Municipal Science and Technology Bureau (2019060002), the Major Scientific Research Project of Wuxi Health Committee (Z202013), Top Talent Support Program for Young and Middle-Aged People of Wuxi Health Committee (HB2020079), Scientific and Technological Development Fund from Wuxi Science and Technology Bureau (Y20212008), Wuxi Municipal Health Commission Scientific Project (T201144), and Jiangsu Geriatrics Clinical Technology Application Research Project (LR2021040).
Primary sponsor	N/A
Secondary sponsor(s)	N/A
Contact for public queries	Bin Su, 13951585359@163.com
Contact for scientific queries	Bin Su, 13951585359@163.com
Public title	Effect of cerebellar vermis iTBS on the balance dysfunction of stroke patients and the brain remodeling mechanism
Scientific title	Effect of cerebellar vermis iTBS on the balance dysfunction of stroke patients and the brain remodeling mechanism
Countries of recruitment	China
Healthy conditions(s) or problem(s) studied	Stroke with hemiparesis, including balance dysfunction
Intervention(s)	Active comparator: iTBS (application of 600 pulses with a frequency of 50 Hz, in a theta-rhythm of 5 Hz for 200 s) will be applied to the cerebellum vermis positioned tangentially to the scalp combined with routine rehabilitation treatment. Sham comparator: Sham stimulus will be applied to same location with a customized sham coil, also combined with routine rehabilitation treatment.
Key inclusion and exclusion criteria	Inclusion criteria: (a) have a diagnosis of stroke confirmed by CT and/or MRI, (b) have their first-ever unilateral ischemic stroke between 1 and 6 months of onset, (c) are 40–75 years old, (d) presented with a balance dysfunction with a Berg score between 0 and 40, (e) can follow simple verbal commands or instructions, and (f) can cooperate in signing informed consent forms. Exclusion criteria: (a) with contraindications involving TMS and fMRI (e.g., intracranial implant, cardiac pacemaker, implanted drug pumps, and pregnancy), (b) with cerebellum or brainstem stroke, (c) with balance dysfunction before stroke, (d) with serious diseases that may contribute to condition progression, (e) with severe deficits in communication or executing commands that prevent cooperation with assessment and treatment, (f) who are taking psychotropic drugs, and (g) who are currently enrolled in other clinical trials.
Study type	Interventional Allocation: randomized intervention model Masking: participants and assessors-blinded Assignment: parallel Primary purpose: treatment
Date of first enrolment	Not yet started
Target sample size	40
Recruitment status	Recruiting
Primary outcome(s)	Berg balance scale
Key secondary outcome(s)	Lower extremity of the Fugl–Meyer assessment; modified Barthel index; regional homogeneity, fractional amplitude of low frequency fluctuation and functional connectivity analyses from rs-fMRI.

### Participants

#### Inclusion Criteria

The inclusion criteria are as follows. Participants who (a) have a diagnosis of stroke confirmed by CT and/or MRI, (b) have their first-ever unilateral ischemic stroke between 1 and 6 months of onset, (c) are 40–75 years old, (d) presented with a balance dysfunction with a Berg score between 0 and 40, (e) can follow simple verbal commands or instructions, and (f) can cooperate in signing informed consent forms.

#### Exclusion Criteria

Participants (a) with contraindications involving TMS and fMRI (e.g., intracranial implant, cardiac pacemaker, implanted drug pumps, and pregnancy), (b) with cerebellum or brainstem stroke, (c) with balance dysfunction before stroke, (d) with serious diseases that may contribute to condition progression, (e) with severe deficits in communication or executing commands that prevent cooperation with assessment and treatment, (f) who are taking psychotropic drugs, and (g) who are currently enrolled in other clinical trials will be excluded from the study.

### Sample Size

The sample size was calculated using a G*power of 3.1.4. The effect size was calculated as 0.28 based on the BBS from a study reported by Koch et al. (2018). To achieve 95% power with an alpha error of 5%, a sample size of 30 patients will be required. Allowing for a dropout rate of 20%, at least 38 patients are necessary. To ensure enough power to detect this typical difference, we will aim to recruit 40 patients (20 patients per group).

### Randomization

**FIGURE 2:**
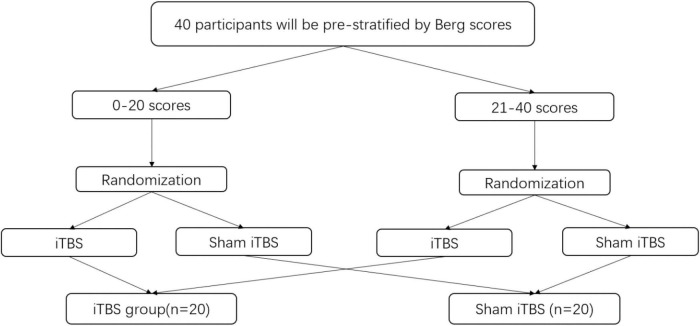
Flow chart of random grouping procedure.

All participants will be prestratified into two subgroups (i.e., poor balance on 0–20 scores vs. acceptable balance on 21–40 scores) on the basis of their balance function to ensure a relatively even distribution between groups (Lisa and Nicol, 2008) (Figure 2). After obtaining informed consent, a random sequence will be generated using the computer, 40 participants will be randomly allocated in a 1:1 ratio to each group and will receive either real or sham iTBS. The advantage of this type of assignment process, which is designed in our study, is that detect effects can be improved especially for studies involving small samples.

### Blind

This study is a participants and assessors-blinded study. Given the nature of the iTBS intervention, blinding the physicians performing the intervention is impossible. The intervention team needs to be separated into blinded physicians performing routine rehabilitation treatment, and unblinded physicians exclusively applying iTBS. However, patients, outcome assessors, research assistants, and statisticians will be blinded. For urgent unblinding, researchers can use sealed envelopes labeled with the patients’ randomization numbers. To maintain the quality of the trial, a patient’s allocation must be unblinded only in exceptional circumstances when knowledge of the actual treatment is essential for the management of the patient.

### Intervention

**FIGURE 3:**
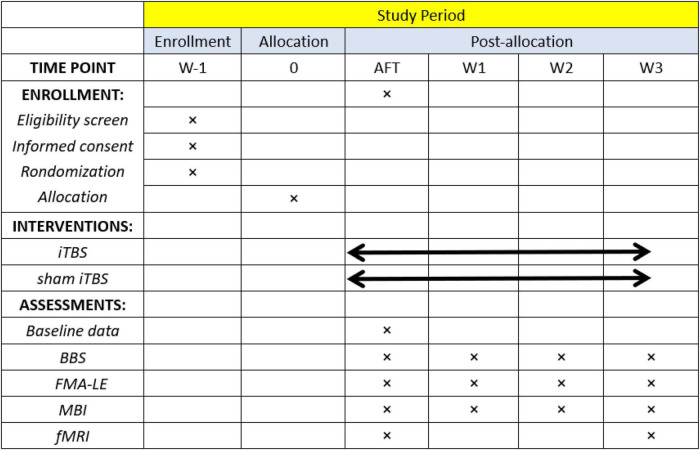
Schedule of participant enrollment, interventions, and assessments. BBS, Berg Balance Scale; FMA-LE, lower extremity of the Fugl–Meyer assessment; MBI, modified Barthel index; fMRI, functional magnetic resonance imaging.

Patients recruited in this study will receive a 60 min routine rehabilitation treatment immediately after completing real or sham iTBS therapy. The rehabilitation program focuses on the exercise designed to promote recovery of balance function, including trunk control, weight bearing, task oriented intervention, and balance training, which will be designed by physicians according to the patient’s balance function. Patients in both groups will be required to attend 15 sessions [one session per day, five times per week (Monday to Friday) for three consecutive weeks]. Enrollment, intervention, and assessment procedures are shown in Figure 3.

Cerebellar iTBS: We use the Magneuro 100 (Vishee Medical Technology Co., Ltd., Nanjing, China) equipped with a double-cone coil (each coil is 70 mm in diameter) to stimulate the cerebellum vermis. iTBS is performed by the physician in accordance with the TMS guidelines (Groppa et al., 2012; Klooster et al., 2016). Before intervention, active motor threshold (AMT) for the tibialis anterior muscle in the unaffected side will be measured. The AMT is defined as the minimum output of stimulation that produced a motor evoked potential (MEP) of >200 μV in at least 5 out of 10 trials with 10% maximal voluntary contraction (Liao et al., 2020). Stimulation will be administered at 80% of AMT. The iTBS paradigm consisting of three pulses at 50 Hz repeated at 5 Hz frequency will be used, with 8-s rest after each 2-s stimulus, generating a total of 600 pulses in 200 s. The coil will be positioned tangentially to the scalp over the cerebellum vermis (at 1 cm below the highest point of the occipital protuberance) (Cattaneo et al., 2014; Tan et al., 2021). Sham iTBS will be performed with a customized sham coil and at the same location, frequency and intensity so that all participants are exposed to a similar clicking noise.

### Outcome Assessment

#### Primary Outcome

The BBS is a clinical test commonly used to assess the patients’ static and dynamic balance performance and can determine whether a patient is at risk of falls with an inter-rater reliability of 0.98 (Maeda et al., 2015; Meseguer-Henarejos et al., 2019). The BBS consists of 14 items, and the score for each item is 0–4 points with an overall score of 0–56 points. Good completion results in high score.

#### Secondary Outcomes

In addition to the BBS scores, FMA-LE, MBI, and rs-fMRI will be measured with the following tests.

Fugl–Meyer assessment of the lower extremity has been widely used in the assessment of the motor function of patients with stroke and has good prediction, reliability, and sensitivity to interventions (Balasubramanian et al., 2016). In addition, the reliability of the FMA scale is >75% between different raters and >88% within the same rater (Hernández et al., 2021). The FMA-LE consists of 17 items, with each item scoring a minimum of 0 and a maximum of 2 points, resulting in a maximum total score of 34 points. A high score results in good motor function of the lower extremity.

The MBI, one of the main scales used to measure the patients’ abilities to perform activities of daily living, has a reliability of 88% in patients with ischemic stroke (Yang et al., 2021). The MBI consists of 10 items, with a full score of 100 points. A high score results in good independence of the patient’s life.

All subjects will undergo rs-fMRI scans. Regional homogeneity (ReHo), fractional amplitude of low-frequency fluctuation (fALFF), and functional connectivity (FC) will be calculated to measure the local and global FC networks related to motion function. All images will be acquired by professional personnel in a 3.0T, 8-channel MRI scanner (Siemens Skyra, Germany). During scanning, subjects will be required to keep awake, breathe quietly, lie flat on the bed, fix their heads to minimize the movement of their heads and other parts, and try not to do any active thinking activity. It will take approximately 40 min to complete the test. Conventional T1 structural image parameters are as follows: repetition time (TR) = 2,000 ms, echo time (TE) = 20 ms, field of view (FOV) = 240 mm × 240 mm, slice thickness = 4 mm, slice spacing = 4 mm, flip angle (FA) = 90°, and 36 slices. Functional images will be acquired using T1-weighted gradient echo planar imaging (EPI) sequence in the axial plane with the following parameters: TR = 2,000 ms, TE = 30 ms, FOV = 240 mm × 240 mm, slice thickness = 4 mm, FA = 90°, and 35 slices.

### Statistical Analysis

#### Clinical Data Analysis

The SPSS (version 22.0, Chicago, IL, United States) statistical software will be used for data analysis. The difference will be considered statistically significant when the *P*-value ≤ 0.05 in two-tailed test. The assumption of normality will be verified by normal probability plots and the Shapiro–Wilk test. Continuous variables with normal distribution will be described as the mean (standard deviation) and analyzed by an independent-samples *t*-test. For abnormally distributed variables, the data will be described as medians with ranges and compared by Mann–Whitney U test (non-parametric tests). Categorical variables will be expressed as frequency (percentage) and Chi-square test or Fisher’s exact test will be used. Repeated measures analysis of variance (ANOVA) will be used to analyze value changes of BBS, FMA-LE, and MBI across four testing time points (weeks 0, 1, 2, and 3). When the interaction is significant, *post hoc* analysis will be conducted, and the Bonferroni adjustment will be used for paired comparisons. Data on clinical scales will be based on per-protocol (PP) and intention-to-treat (ITT) analyses (Sanchez and Xun, 2010). The PP is usually defined as subjects who complete at least 80% of the treatment protocol (Zhao et al., 2021). An ITT analysis include all the patients recruited in the study, irrespective of whether they completed the study period or not. The missing values will be imputed using a multiple imputation method.

#### Functional Magnetic Resonance Imaging Data Analysis

On the MATLAB R2013b (Mathworks, Natick, MA, United States) platform, the statistical parametric graph software (SPM12.0^1^) and data processing assistant for rs-fMRI software (DPARSF^2^) will be used for data processing and analysis. DPARSF is a SPM-based rs-fMRI data processing software (Yan et al., 2016). Preprocessing steps include (Lv et al., 2018; Ll et al., 2020): (a) removing the first 10–20 time points and slice timing, (b) head movement correction (head movement exceeding 2 mm in any direction or head rotation exceeding 2° will be ruled out), (c) normalization (in accordance with the Montreal Neurological Institute EPI template, each voxel is resampled to 3 mm × 3 mm × 3 mm), (d) delinear drift, (e) removal covariate (confounding effects of head movement parameters, white matter, and cerebrospinal fluid signals), (f) filter (0.01–0.1 Hz), and (g) smooth (8 mm full-width half-maximum Gaussian). Then, the ReHo, fALFF, and FC values will be calculated. The ReHo analysis is a voxel-based measurement that calculates the Kendall coefficient of concordance between a voxel and its surrounding 26 voxels, which is the ReHo value of that voxel (Zang et al., 2004). The ReHo analysis determines the synchrony of adjacent regions, and high values indicate high consistency of regional brain activity. The ALFF measurement is the root mean square of the power spectrum of the BOLD signal in the range of 0.01–0.1 Hz for each voxel. Afterward, the ALFF value of each voxel divided by the full-band ALFF sum is the fALFF value, which can reflect the strength of brain self-activity (Toole et al., 2005). FC was examined by using a seed voxel correlation approach. The cerebellar vermis will be selected as the seed region based on the stimulation target. In SPM12, after bandpass filtering and linear-trend removal, a reference time series for the seed will be extracted by averaging the fMRI time series of voxels. Then, correlations will be computed between the seed reference and the rest of the brain in a voxelwise manner. Finally, an individual relativity value (*r*-value) map will be created, and the correlation coefficients in each voxel will be transformed to *z* values by using Fisher’s *r*-to-*z* transformation to improve normality before performing random effect *t*-tests. The AlphaSim will be used for correction to obtain the adjustment statistical threshold value of *P* < 0.05. Data will be analyzed by the independent-samples (between-group comparison) or paired (within-group comparison) *t*-test.

### Safety

Operation procedures will strictly follow the latest safety guidelines for TMS (Rossi et al., 2009), and participants will be screened strictly in accordance with the inclusion and exclusion criteria to minimize the risk of adverse event. Common adverse events of TMS include (but are not limited to) headache, dizziness, tinnitus, and seizures. If mild headache, discomfort caused by noise, and scalp irritation occur, they are usually self-healing and transient. Hence, before testing or intervention, the patient should be informed of the risks in advance. Seizures, the most severe TMS-related adverse event with a crude risk of approximately 0.02% (Rossi et al., 2009), are expected to occur only during or immediately after stimulation. In addition, any adverse event occurring within 24 h after MRI will be reported. If a serious incident occurs, it will be immediately reported to the principal researcher and ethics committee. The ethics committee will assess whether the patient should receive further treatment. If the patients suffer from syncope, seizures, and other conditions that may affect the subject’s health during the treatment, we will stop the assigned intervention. Once the participants are injured during this trial, they will be compensated. All adverse events from TMS will be reported by the researcher and recorded on the case report form. The researchers will record the date, duration, and severity of adverse events.

### Patient and Public Involvement

During the trial, patients will not participate in the design, implementation, or outcome assessment of the study. However, we plan to publicize our research proposal to participants and the public through the official website of the hospital (such as WeChat official account) and lectures at major academic conferences.

### Ethics and Dissemination

The study has been approved by the Medical Research Ethics Committee of Wuxi Mental Health Center (Wuxi Tongren Rehabilitation Hospital). Before entering the study, all participants will be informed that their participation are entirely voluntary and that they can withdraw from the trial at any time without consequence. During the trial, the welfare and rights of participants should be satisfied, and the privacy of patients should be guaranteed. Any change in the key aspects of the protocol, such as inclusion criteria, observation indicators, and statistical analysis, will be submitted to the Medical Research Ethics Committee. Results will be presented at conferences and submitted to peer-reviewed journals.

### Research Quality Assurance and Management

Researchers should perform their respective responsibilities. Including the following contents:

1. The principal researcher establishes unified experimental test indices, standard operating procedures, and quality control procedures.
2. Researchers participating in our trials must have professional expertise and the ability to conduct clinical trials. Moreover, invariability of personnel involving in this research should be guaranteed.
3. The interveners and evaluators need to participate in standardized training before the trial, they should adopt standard operating procedures to ensure the quality of clinical trials.
4. The principal researcher shall appoint an inspector to ensure that the rights and interests of participants are protected, that the trial follows approved protocols, and that reports are accurate.

## Discussion

This 3-week randomized controlled clinical trial aims to study whether cerebellar vermis iTBS can improve balance function in patients with stroke. In this perspective of experiment design, our study puts forward some methodological concerns that should be discussed, including the large variation in balance function among enrolled patients. Therefore, a stratification approach will be adopted in this study to divide all participants into two subgroups in accordance with the BBS. In addition, we set a sham stimulation group to eliminate the placebo effect of iTBS intervention. After careful consideration of all factors, including the feasibility of the study and the treatment time pattern of the hospital where the experiment will be conducted, we set sessions from Monday to Friday for 3 weeks. Also, outcome will be evaluated at pre-intervention, the end of first, second, and third week of post-intervention. We want to compare the efficacy at different time points, and to explore the earliest time when the treatment can take effect. Our study will use rs-fMRI rather than task state fMRI for measuring the iTBS-induced effects, although task state fMRI can reflect the fMRI images of the brain when performing specific tasks. What we considering is that our subjects are post-stroke patients with motor dysfunction, there may be great differences in the quality of tasks completed by stroke patients, which may affect the results.

In this study, we expect that the cerebellar vermis iTBS can promote the recovery of balance function after stroke. This hypothesis is based on the following evidence.

Maintaining balance depends upon a complex coordination and integration of multiple systems in the body, including the visual system, vestibular system, and auditory system. As pointed out in fMRI research, the cerebellar vermis and the visual network have a strong functional connection (Kellermann et al., 2012; Li et al., 2012). Recent evidence displayed that the vermis coordinates eye and body movements, and provides visual and auditory input related to balance (Fujita et al., 2020). In addition, studies have shown that the vermis participates in regulating vestibular system and maintaining head position (Lam et al., 2016). Balance is best maintained when all inputs are integrated together. Therefore, the cerebellar vermis plays an essential role in the active maintenance of body balance.

In addition, the process of maintaining balance involves a close cooperation between the cerebellum and the cerebrum. These interconnections are facilitated by white-matter tracts with the following connective pathways: the cortico-ponto-cerebellar pathway and cerebello-thalamo-cortical (CTC) pathway (Iwata and Ugawa, 2005; Opie and Semmler, 2020), which have been proven to be involved in motor coordination (Spampinato et al., 2020). As pointed out in an animal experiment, the reduction of ipsilateral cerebral cortical activity can lead to the reduction of contralateral cerebellar activity and metabolism (Baek et al., 2020). Previous studies similarly confirmed that the cerebellum establishes strong anatomical and functional connections with the M1 area through the cerebellum–thalamic–M1 circuit (Maurer et al., 2016). The cerebellum has three functional areas, the spinocerebellum, the cerebrocerebellum and the vestibulocerebellum. The vermis classically belongs to the “spinocerebellum” and to receive somatic sensory input from ascending spinal pathways. Cattaneo et al. (2014) pointed out that compared to cerebellar hemispheres, cerebellar vermis takes a more prominent role in continuous visual processing in visual feedback loops. Interestingly, in Coffman et al.’s (2011) study, the rabies virus was injected into the lobule of the vermis, and retrograde transneuronal transport of the virus was used to define disynaptic inputs to it. It was found that large numbers of neurons in the primary motor cortex and other motor areas on the medial wall of the hemisphere project to the vermis. This implicates that there is potential connection between cerebellum vermis and motor area of cerebral cortex. Therefore, iTBS may improve balance by regulating the excitability of the cerebellar vermis, then promoting excitability changes in motor areas of the cerebral cortex.

In current studies, the depth of coil stimulation is an important factor affecting the efficacy of rTMS. Recent studies showed that an eight-figure coil has mostly been used for iTBS (Li et al., 2018). This coil is characterized by small stimulation area, shallow stimulation depth, and good focusing. The range of the magnetic field induced by rTMS attenuates with increasing distance from the scalp surface (Deng et al., 2013). However, the cerebellum is located in the posterior fossa and covered by the tentorium cerebellum, which is deeper from the skull. Previous studies found that the double-cone coil can achieve deeper stimulation effects (Deng et al., 2013) and generate stronger electric field than the eight-figure coil (Mai and Ueno, 2017), confirming good reliability and validity. In addition, Hardwick et al. (2014) showed that the double-cone coil causes the most evident cerebellar brain effect in magnetic stimulation with the same parameters compared with the eight-figure and airfoil coils, suggesting that the double-cone coil may be suitable for studies of TMS over the cerebellum.

In our trial, the location of cerebellar vermis is consistent with similar literatures (Cattaneo et al., 2014; Tan et al., 2021), In Cattaneo’s study, the accuracy of this localization method was further verified by using a neuronavigation system in some subjects for whom structural MRIs were available. The results shown that the scalp coordinate 1 cm below the inion indeed corresponded to the cerebellar vermis (Cattaneo et al., 2014). This research is the first study to explore the effect of cerebellar vermis iTBS on balance dysfunction after stroke by using the double-cone coil. This protocol proposes a rigorous randomized parallel controlled trial design that meets the methodological requirements of full concealment, randomization, and placebo control.

Intermittent theta burst stimulation has the advantages of short stimulation time, easy promotion, and relatively easy application. We hope that the results of this study can shed light on this potential effect. We believe that the current protocol and our results will have important implications for the treatment of post-stroke patients with balance dysfunction.

## Ethics Statement

The studies involving human participants were reviewed and approved by the Medical Research Ethics Committee of Wuxi Mental Health Center (Wuxi Tongren Rehabilitation Hospital) (No. WXMHCIRB2021LLky132): Effect of cerebellar vermis iTBS on the balance dysfunction of stroke patients and the brain remodeling mechanism. The patients/participants provided their written informed consent to participate in this study.

## Author Contributions

GH designed the trial protocol, with scientific insight and contributions from YS and BS. JY prepared the human research ethics. LZ provided iTBS and routine rehabilitation training programs. LZ and CL were involved in the overall research design and selection of outcome measure. CR calculated the sample size and designed the statistical analysis plan. LW and GH prepared the first draft of this manuscript and it was reviewed by YS and BS. LW performed the revisions. All authors read and approved the final manuscript.

## Conflict of Interest

The authors declare that the research was conducted in the absence of any commercial or financial relationships that could be construed as a potential conflict of interest.

## Publisher’s Note

All claims expressed in this article are solely those of the authors and do not necessarily represent those of their affiliated organizations, or those of the publisher, the editors and the reviewers. Any product that may be evaluated in this article, or claim that may be made by its manufacturer, is not guaranteed or endorsed by the publisher.

## Footnotes

1
http://www.fil.ion.ucl.ac.uk/spm/

2
http://rfmri.org/dpabi
